# Ultrasonic bone age fractionates cognitive abilities in adolescence

**DOI:** 10.1038/s41598-022-09329-z

**Published:** 2022-03-29

**Authors:** Ilona Kovács, Kristóf Kovács, Patrícia Gerván, Katinka Utczás, Gyöngyi Oláh, Zsófia Tróznai, Andrea Berencsi, Hanna Szakács, Ferenc Gombos

**Affiliations:** 1 Laboratory for Psychological Research, Pázmány Péter Catholic University, 1 Mikszáth sq., 1088 Budapest, Hungary; 2 Adolescent Development Research Group, Hungarian Academy of Sciences-Pázmány Péter Catholic University, 1088 Budapest, Hungary; 3 Institute of Cognitive Neuroscience and Psychology, Res. Centre for Natural Sciences, 1117 Budapest, Hungary; 4 Institute of Psychology, ELTE Eötvös Loránd University, 1075 Budapest, Hungary; 5 Research Centre for Sport Physiology, University of Physical Education, 1123 Budapest, Hungary; 6 Institute for the Methodology of Special Needs Education and Rehabilitation, Bárczi Gusztáv Faculty of Special Needs Education, Eötvös Loránd University, 1097 Budapest, Hungary

**Keywords:** Human behaviour, Intelligence

## Abstract

Adolescent development is not only shaped by the mere passing of time and accumulating experience, but it also depends on pubertal timing and the cascade of maturational processes orchestrated by gonadal hormones. Although individual variability in puberty onset confounds adolescent studies, it has not been efficiently controlled for. Here we introduce ultrasonic bone age assessment to estimate biological maturity and disentangle the independent effects of chronological and biological age on adolescent cognitive abilities. Comparing cognitive performance of female participants with different skeletal maturity we uncover the impact of biological age on both IQ and specific abilities. We find that biological age has a selective effect on abilities: more mature individuals within the same age group have higher working memory capacity and processing speed, while those with higher chronological age have better verbal abilities, independently of their maturity. Based on our findings, bone age is a promising biomarker of adolescent maturity.

## Introduction

Entering a classroom full of first year high-school students is a peculiar experience as we meet pupils who are of the same age, but extremely diverse in terms of stature, behavioural features, social and emotional outlook and even cognitive abilities. This is because adolescent growth and development are not only shaped by the mere passing of time, but they also depend on the timing of puberty onset, which has a large variability across individuals^1–3^. After the onset of puberty, musculoskeletal, reproductive and neurodevelopmental systems of a child start to transform into those of an adult during a period that is extremely malleable but also vulnerable. Our current knowledge is limited regarding the correspondence between physical and psychological maturity in adolescence. Nevertheless, we can hypothesize that apparent changes in physique reflecting hormonal progress are accompanied by the structural remodelling of the brain^4–7^ and followed by functional advancement^8–10^.

Although the variability in the onset of puberty results in discrepancies with respect to developmental trajectories^1,11–13^, such variability has not been very efficiently controlled for in adolescent research^1,4,12^. Additionally, the methods of assessment are mostly outdated, and the dissociation of chronological and biological age has not been addressed clearly. The gold-standard has been the Tanner Scale^14–16^, which is based on bodily features such as breast and testicle sizes, and other secondary sex characteristics visually assessed by a paediatrician. This method is very subjective, it might be unsettling for the subject, and it does not seem to represent contemporary nutritional conditions and secular trends of human growth^3,13,15,16^. The Tanner Scale is based on a post-war longitudinal study carried out in the Harpenden children’s home near London, between 1949 and 1971^14,16^. The Harpenden Growth Study^14,17^ involved neglected, many times orphaned children whose participation would not only raise a number of ethical issues today^16^, but it is highly questionable whether the Tanner Scale can serve as a reliable and valid standard of developmental stages in the present^15,16^.

We believe that more objective indicators of biological age, such as gonadal hormone levels^18^, metabolic markers^19^, genetic indicators^19^, skeletal^20,21^ or even dental^22^ maturity assessments might be better alternatives to replace Tanner staging in developmental research and serve as biomarkers of pubertal onset and progress. The selection of a useful marker needs to be guided by both scientific precision and reliability, and by minimizing risk and burden for participants. While, e.g., gonadal hormone levels might appear as an apparently good measure of pubertal maturity, those are invasive (require multiple blood tests) and less reliable during the mid-pubertal stages^18^. The challenge is to identify an indicator that is correlated with adolescent hormonal development, minimally invasive or non-invasive, and, at the same time, is also sensitive enough to identify teenagers who are either advanced or delayed compared to their peers.

Bone age has been widely used in paediatric practice^23,24^, and it is generally based on hand and wrist X-ray radiographs assessing the size and geometry of the epiphysis and fusion of the epiphysis and diaphysis. It has been shown that pubertal hormone levels are correlated more with skeletal system development and bone age than with chronological age^25–27^. This raises the possibility that bone age might be a useful proxy of pubertal maturity. The prospect of a bone age-based pubertal maturity assessment paradigm for research is enhanced by a non-invasive ultrasonic version of this method that scans the ossification of the wrist region to estimate bone age^28^. We have shown that ultrasonic bone age estimations of the wrist very strongly correlate with X-ray estimations^29^ (r > 0.95), and therefore provide a promising tool to replace subjective, invasive, risky, or costly assessments of maturity. The association between menarche age and maturity assessed by ultrasonic bone age in the current study (see Supplementary Fig. S1) is also reassuring that bone age might be a proper proxy of maturity during the teenage years. Ultrasonic bone age assessment is harmless, extremely fast, relatively inexpensive, and portable. In addition to laboratory studies, it might be ideal also for large cohort- and longitudinal studies.

**Figure 1:**
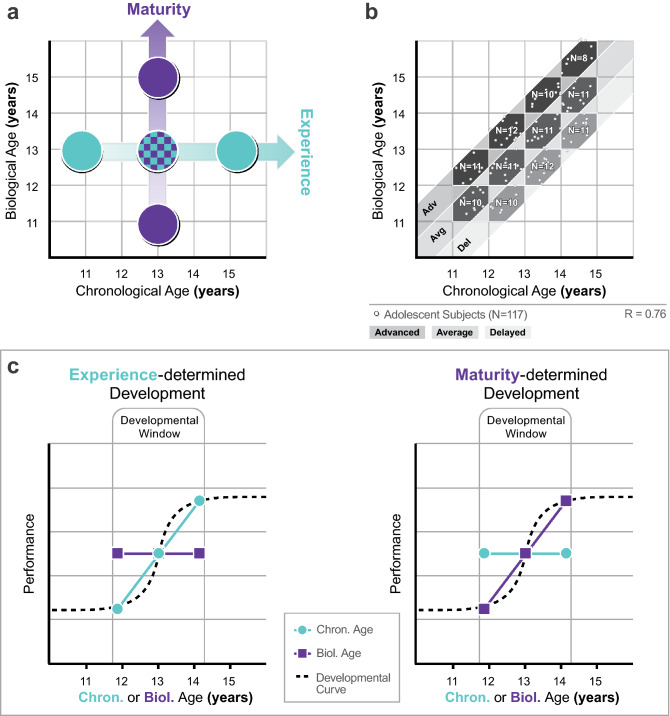
Dissociating the effects of experience and maturation in adolescent development. (**a**) Comparing cognitive performance of participants with different biological age (BA) within the same chronological age-group can reveal the impact of biological age (vertical ‘Maturity’ axis) independently of chronological age (CA), while the comparison of different CA participants with the same maturity level reveals the impact of CA independently of maturation (horizontal “Experience” axis). (**b**) In order to dissociate the impact of BA and CA we identified cohorts of participants with advanced, average, and delayed maturity using bone age assessment within 11 and 15 years of CA. Each 1-year-wide hexagonal bin consisted of 8–12 subjects (white dots within the bins). **c** Predictions: Vertical axes indicate development in any cognitive domain. Horizontal axes express age (CA or BA). Important developmental events occur within a “Developmental window” where performance radically changes approaching adult levels. In the extreme case of experience-determined development, we expect to see a significant improvement of performance between different CA groups (matched for BA), while performance will remain the same across different BA groups (matched for CA). In the other extreme case where development is solely maturity-dependent, we expect improvement across different BA groups, and the lack of change across different CA groups. The final version of the figure was generated by Adobe Illustrator CC 23.02.2. (64-bit).

In this study, biological age (BA) of participants is assessed by evaluating the acoustic parameters of the left wrist, establishing bone age for each participant (“Methods/Procedure”). In order to estimate the independent effects of BA and chronological age (CA) on development we introduced the paradigm presented in Fig. 1. Preselected participants along the Maturity axis of Fig. 1a have the same CA and different BAs, therefore, any differences in their cognitive performance will be accounted for exclusively by BA without the influence of CA. And vice versa, performance of participants with different CAs along the Experience axis will only be determined by CA as there is no variability in BA. Aiming at this clear-cut dissociation, we identified cohorts of adolescent participants with average (BA = CA), advanced (BA > CA) and delayed pace of skeletal maturation (BA < CA, Fig. 1b) reflecting their pubertal maturity levels. This design allows for predictions with respect to developmental scenarios where the amount of experience (CA) or the level of maturity (BA) might have different contributions to the studied psychological function (Fig. 1c).

Predominantly experience-determined development will result in a steep developmental function comparing subjects along the Experience axis of Fig. 1a, while more maturity-determined development will generate a steep function comparing participants along the Maturity axis. It is important to note that the “developmental window” within which a particular psychological function significantly improves might shift across different age-bands depending on the timing of the maturation of the underlying cortical structures. To determine narrower age-bands where the impact of BA/CA might be prominent we generated a number of distinct BA-CA bins shown in Fig. 1b (for a more detailed definition of BA-CA bins see “Methods/Participants”).

The paradigm introduced in Fig. 1 provides a tool to study development in any particular domain. We chose to assess the relationship between cognitive development and skeletal maturity. There has been very limited research in this area, and it is mostly related to extreme skeletal maturational lags that can negatively impact intellectual development^30,31^. Our purpose is to assess the relationship between intellectual development and pubertal maturity within the typical range of developmental parameters. In the lack of extended previous research, we opted for the mapping of different broad cognitive abilities measured by the Wechsler Intelligence Scale for Children.

Traditionally, age effects have been among the primary means towards fractionating human cognitive abilities. In particular, the distinction between fluid and crystallized intelligence, the forerunner of one of the most respected psychological theories on the structure of cognitive abilities, the Cattell–Horn–Carroll (CHC) model^32^, was to a large extent based on the finding that fluid reasoning (the ability to solve novel problems) show developmental patterns very different from crystallized intelligence (the ability to use already acquired skills and knowledge). Different trajectories of cognitive development provide another means towards dissociating aspects of cognition. For instance, executive functions keep maturing well into the late teenage years, perhaps even until the early twenties^9,10,33^. Performance on different ability tests indeed have very different peaks in life: while tests of memory or processing speed peak as early as the end of teenage years, performance on verbal tests such as those measuring vocabulary or comprehension peaks at near 50 years of age, indicating that experience matters much more for verbal comprehension than for other abilities^34^.

To explore the independent and potentially selective effects of maturation and experience on the cognitive development of a preselected cohort of adolescents (Fig. 1b), we administered 11 subtests from the Hungarian adaptation of the Wechsler Intelligence Scale for Children IV—WISC-IV^35^. According to the hypotheses presented in Fig. 1c, we expected to see a pattern in the results where certain cognitive abilities are more determined by BA, and others by CA. In particular, according to previous results^34,36^ and the theory of fluid and crystallized intelligence^32,37^, we expected verbal abilities to be more influenced by chronological age (probably reflecting the amount of schooling) than non-verbal abilities.

Indeed, our most striking finding is that while verbal abilities are independently influenced by experience (CA has a significant effect when BA is partialled out), the opposite is the case for working memory and processing speed (BA has a significant effect when CA is controlled for). That is, in adolescents of the exact same age, those who are more mature have higher memory capacity and mental speed, while among adolescents at the same level of maturation, those with a higher CA have better verbal abilities.

## Results

Following the assessment of their bone age, participants (11- to 15-year-old, N = 117, see “Methods” and Fig. 1b) were administered 11 WISC-IV subtests (see “Methods/Procedure”). We only tested female participants in order to control for the different maturational trajectories resulting from a different hormonal composition between the two sexes^13,38–40^. To control for the impact of different socio-economic and socio-cultural backgrounds^9,39,41^, we restricted the participant pool to only include pupils arriving from top-level high schools (see “Methods/Participants”).

Since we purported to study the effect of age-related factors on test performance across age groups, age-standardized scores were not suitable. Therefore, we used unstandardized raw scores, in percentage, for each subtest. Overall performance was calculated by averaging the 11 subtests. We also calculated scores for each of the broad abilities reflected by the indices of the WISC-IV (Verbal Comprehension, Perceptual Reasoning, Working Memory, Processing Speed) by averaging the subtest scores corresponding to each. See Supplementary Tables S1 and S2 for descriptive statistics and basic correlations.

### Overall cognitive performance is determined both by biological and chronological age

**Figure 2:**
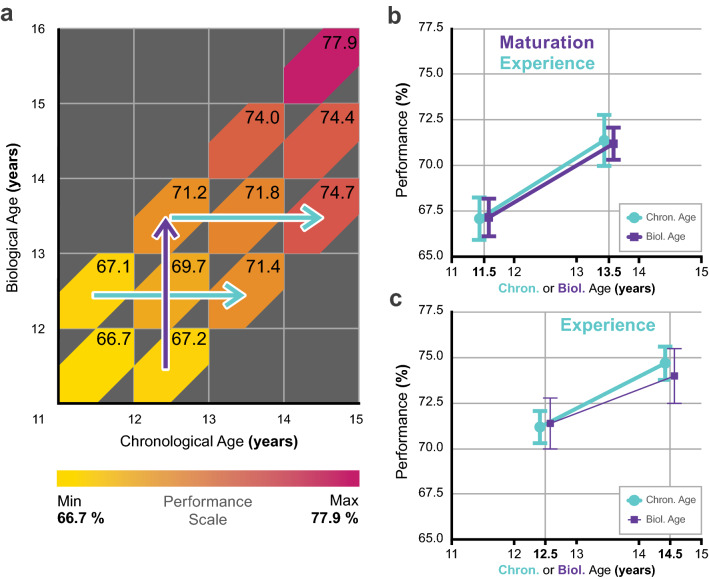
WISC-IV overall performance as a function of biological and chronological age. (**a**) Average WISC-IV overall performance (based on raw scores) was calculated for each bin as a % of possible maximum performance described in Fig. 1b and expressed on a colour scale (Performance scale). Higher performance is indicated by darker shades of red. Numbers within each coloured bin express performance in % of possible maximum performance. Purple arrow indicates bins where a significant biological age effect is present. Blue arrow indicates bins where a significant chronological age effect is present. (**b**) Detailed performance within the 11–14 years age-range where significant BA and CA effects according to one-way ANOVA are present (significant developmental changes are represented by bold lines). Both experience and maturation significantly determine performance. **c** Detailed performance within the 12–15 years age-range where significant chronological age-effect is present. Performance is determined by experience. Error bars show 1SE. The final version of the figure was generated by Adobe Illustrator CC 23.02.2 (64-bit).

In order to obtain a comprehensive perspective on mental abilities first, we calculated and averaged the overall performance of participants for each BA and CA defined 1-year bin of Fig. 1b. Overall performance on WISC-IV for each bin is plotted in Fig. 2a. Unsurprisingly, there is increasing performance throughout the entire CA range as adolescents’ cognitive development takes place (remember, the performance measure reflects absolute performance, unlike age-standardised scores such as IQ). A more fascinating trend, however, is the increasing performance as a function of BA. It appears that participants with the same CA but with more advanced bone maturity achieve higher scores on the IQ test!

Pearson correlation was calculated between overall WISC performance and BA/CA within each 1-year wide BA or CA group (see Supplementary Table S6 online). The correlations were Holm–Bonferroni corrected for multiple comparisons. We used one-way ANOVA to look for significant differences between the lower and upper bins within the particular BA/CA age-band with significant correlations (see Supplementary Table S7 online). Coloured arrows in Fig. 2a indicate those BA/CA age-bands where significant differences were found. For the comprehensive understanding of the statistical analyses, and their sequential steps, please see “Methods/Statistics”.

The two axes in Fig. 1a provide a clear dissociation of maturity (BA) and experience (CA) effects. Since the developmental window (Fig. 1c) for cognitive development might shift across different age ranges depending on timing of the maturation of the underlying cortical structures, it is important to analyse the results within narrower age-ranges and identify the age where the impact of CA/BA is most prominent. Figure 2b presents detailed analysis within a developmental window of 11 to 14 years of CA/BA with CA comparisons for BA being constant at 12.5, and BA comparisons for CA being constant at 12.5. Apparently, both CA (67.1% ± 1.2 SE vs. 71.4% ± 1.4 SE; p = 0.029) and BA (67.2% ± 1.0 SE vs. 71.2% ± 0.9 SE; p = 0.007) significantly determine cognitive performance within this CA/BA range. In terms of the predictions of Fig. 1c, development within this window is determined both by experience and maturation. Moving to the next developmental window of 12 to 15 years of CA/BA (Fig. 2c) there seems to be a similarly strong CA effect (71.2% ± 0.9 SE vs. 74.7% ± 0.9 SE; p = 0.011), however, the effect of BA is no longer significant (71.4% ± 1.4 SE vs. 74.0% ± 1.5 SE; p = 0.210). Based on these findings, BA has the strongest effect on overall performance between the ages of 12–13 years. Interestingly, the centre of this window is very close to the average menarche age (12.1 ± 0.9 SD years) of our participants.

In addition to the graphical analysis of Fig. 2, partial correlation analysis was also performed in the whole group to disentangle the independent effect of chronological age and biological age. We performed bootstrapping on 1000 samples to obtain confidence intervals for the partial correlation coefficients.

Additionally, multiple linear regression was calculated to predict Overall performance based on bone age (BA) and chronological age (CA). A significant regression equation was found (F(2,114) = 49.444, p < 0.001, R^2^ = 0.465). Both BA and CA were significant predictors of Overall performance.

The results are summarized in Tables 1 and 2 (results for the 11 subtests are summarized in Supplementary Tables S3 and S4). These analyses demonstrated that the independent effect of both BA and CA on overall performance is significant, substantial, and of very similar magnitude (pr = 0.298, p < 0.01 and 0.320, p < 0.001, respectively; B = 0.016, p < 0.01, and B = 0.016, p < 0.001, respectively).

**Table 1:** Partial correlations between chronological/biological age and WISC-IV overall performance, and performance on the four factors.

	Chronological age	95CI	p	Biological age	95CI	p
Overall performance	*0.295*	*0.130 to 0.449*	*0.001*	**0.328**	**0.142–0.488**	**< 0.001**
Verbal comprehension	**0.320**	**0.159 to 0.470**	**< 0.001**	0.060	−0.141 to 0.252	0.524
Perceptual reasoning	0.152	− 0.040 to 0.343	0.104	0.075	− 0.110 to 0.262	0.425
Working memory	− 0.080	− 0.272 to 0.118	0.395	**0.360**	**0.201 to 0.504**	**< 0.001**
Processing Speed	0.173	− 0.037 to 0.380	0.063	**0.327**	**0.166 to 0.477**	**< 0.001**

Italicised values: p < 0.01, Bold values: p < 0.001.

Partial correlations between chronological age/biological age and WISC-IV broad abilities and overall performance. The correlations represent the *independent* effect of chronological age and biological age, with the effect of the other kind of age controlled for. 95CI = 95% confidence interval.

**Table 2:** Multiple linear regressions for the effects of chronological/biological age on WISC-IV overall performance and on the four factors.

	B	95CI	Beta	p	Model parameters
**Full scale**					
Chronological age	*0.016*	*0.006 to 0.025*	*0.342*	*0.001*	F(2,114) = 49.444, p < 0.001 R = 0.682 R^2^ = 0.465
Biological age	**0.016**	**0.007 to 0.024**	**0.386**	**< 0.001**	
**Verbal comprehension**					
Chronological age	**0.034**	**0.015 to 0.052**	**0.443**	**< 0.001**	F(2,114) = 19.433, p < 0.001 R = 0.504 R^2^ = 0.254
Biological age	0.005	− 0.011to 0.022	0.079	0.524	
**Perceptual reasoning**					
Chronological age	0.013	− 0.003 to 0.030	0.222	0.104	F(2,114) = 6.113, p < 0.01 R = 0.311 R^2^ = 0.097
Biological age	0.006	− 0.009 to 0.021	0.108	0.425	
**Working memory**					
Chronological age	− 0.008	− 0.025 to 0.010	− 0.108	0.395	F(2,114) = 14.310 p < 0.001 R = 0.448 R^2^ = 0.201
Biological age	**0.033**	**0.017 to 0.048**	**0.524**	**< 0.001**	
**Processing speed**					
Chronological age	0.014	− 0.001 to 0.030	0.214	0.063	F(2,114) = 31.733, p < 0.001 R = 0.598 R^2^ = 0.358
Biological age	**0.026**	**0.012 to 0.039**	**0.421**	**< 0.001**	

Italicised values: p < 0.01, Bold values: p < 0.001.

Multiple linear regressions for chronological age/biological age on WISC-IV broad abilities and overall performance. *B* unstandardised coefficients, *beta* standardised coefficients, *95CI* 95% confidence interval.

As discussed in the next section, this similar sized effect is not identical across specific cognitive abilities. On the contrary: the effect of BA and CA is strongly dissociable.

Since multicollinearity can result in artifacts in multiple regression in the case of strongly correlated independent variables, and in our sample the correlation between BA and CA was strong (r = 0.76), we have performed relative weight analysis (RWA, see Supplementary Table S5 online), which addresses exactly this issue. The results are summarized in Supplementary Table S5. In the case of overall performance BA and CA have almost identical contributions (51.5% vs 48.5%, respectively) to the variance explained (R^2^) in the multiple regression model.

### Selective effects of biological and chronological age on specific cognitive abilities

Similarly to the analysis of overall performance, we first calculated partial correlations to dissociate the effect of maturity (BA) and experience (CA) on specific abilities reflected by the four indices of the WISC.

Second, four individual multiple linear regressions were calculated to predict specific cognitive abilities (Verbal Comprehension (VC), Perceptual Reasoning (PR), Working Memory (WM) and Processing Speed (PS)) based on bone age (BA) and chronological age (CA). A significant regression equation was found in every instance (VC: F(2,114) = 19.433, p < 0.001, R^2^ = 0.254; PR: F(2,114) = 6.113, p < 0.01, R^2^ = 0.097; WM: F(2,114) = 14.310 p < 0.001, R^2^ = 0.201; PS: F(2,114) = 31.733, p < 0.001, R^2^ = 0.358).

The partial correlation and multiple regression analyses yielded very similar results. CA has a significant effect on Verbal Comprehension, independently of BA (pr = 0.320, p < 0.001; B = 0.034, p < 0.001), while BA does not have a significant effect independent of CA. For Working Memory and Processing Speed we identified the opposite pattern: BA has a significant effect independent of CA (pr = 0.360, p < 0.001 and 0.327, p < 0.001, respectively; B = 0.033, p < 0.001, and B = 0.026, p < 0.001, respectively). Neither BA nor CA was a significant predictor of Perceptual reasoning.

The results are summarized in Tables 1 and 2 (results for the 11 subtests are summarized in Supplementary Tables S3 and S4).

A comparison of the partial correlations in Table 1 with the Pearson correlations in Supplementary Table S2 reveals that the independent effect of BA and CA do not manifest themselves in the latter. This should not come as a surprise given that BA and CA share most of their variance. Therefore, it is all the more remarkable that substantial independent effects could be statistically identified, and the effect of BA and CA could be dissociated for specific cognitive abilities.

Importantly, these effects are not driven by unique effects of individual subtests: as it is apparent from Supplementary Tables S3 and S4, the independent effect of BA and CA is uniform across each broad cognitive ability. That is, in each subtest that contributes to a given index score, the same significant independent effect was identified as in the index score itself that reflects a particular specific ability. This demonstrates the construct-level effect of BA and CA: they do not simply influence performance on individual subtests, but rather on specific abilities.

There was no significant effect of BA or CA on Perceptual Reasoning.

Similarly to results regarding overall performance, we have performed relative weight analysis (RWA, see Supplementary Table S5 online) to address the multicollinearity of the predictors. In the case of Working Memory the relative weight of BA and CA was 78.4% and 21.6%, respectively. In the case of Verbal Comprehension, the proportions lean towards CA: the relative weight of BA and CA was 33.8% and 66.2%, respectively. For Processing Speed and Perceptual Reasoning there is a smaller difference between the contribution of BA and CA to the variance explained by the predictors: PS—58% and 42%, respectively, PR—41.6% and 58.4%, respectively. The difference between relative weights was only significant in the case of Working Memory.

### Age-specific maturity effects on specific cognitive abilities

**Figure 3:**
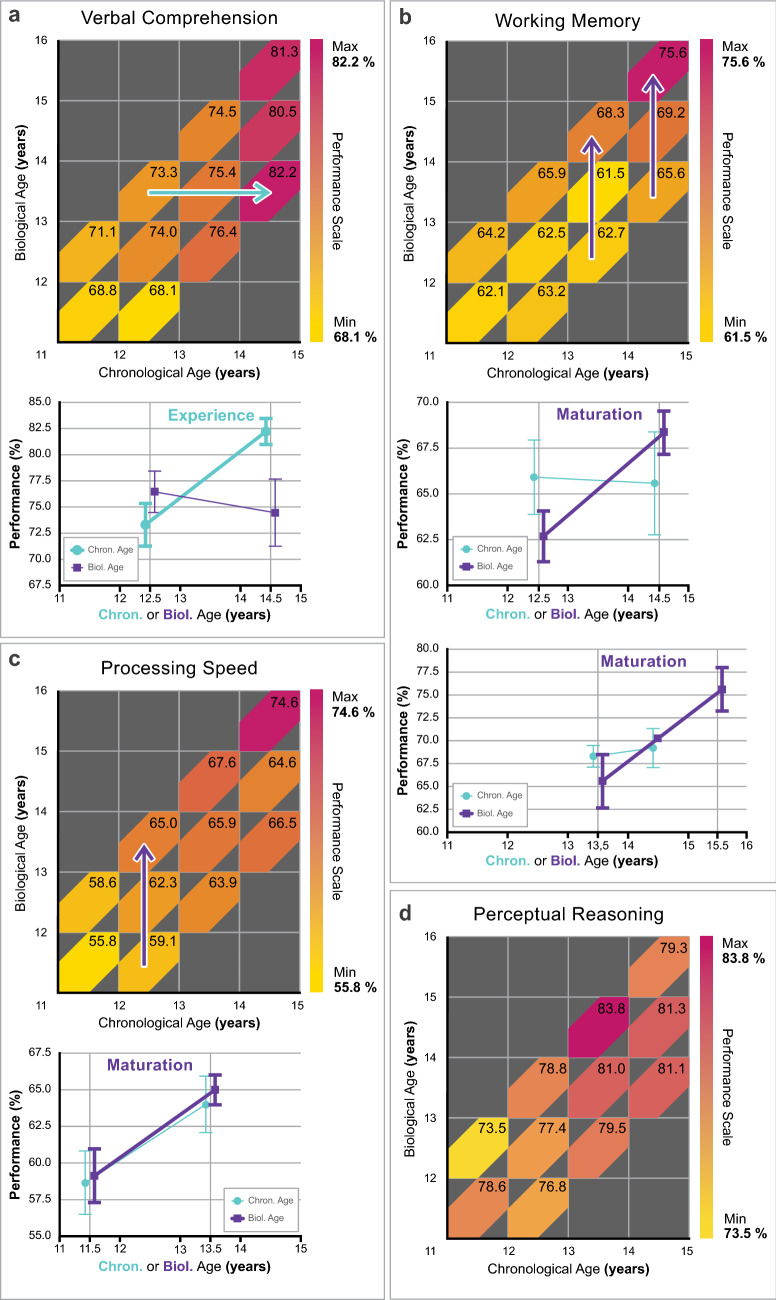
Performance in specific abilities as a function of biological and chronological age. Average performance (based on raw scores) for the four factors of WISC-IV was calculated as a % of maximum possible performance for each bin described in Fig. 1b and expressed on a colour scale (Performance scale). Higher performance is indicated by darker shades of red. Numbers within each coloured bin express performance in % of maximum performance. Purple arrow indicates bins where a significant biological age effect is present. Blue arrow indicates bins where a significant chronological age effect is present. Line graphs show detailed performance within specific age-ranges where there are significant BA and/or CA effects according to one-way ANOVA (significant BA and/or CA effects are represented by bold lines). **a** Verbal Comprehension is determined by chronological age, significant performance changes are only observed along the ‘Experience’ axis within the age-range of 12.5–14.5 years. **b** Working Memory is determined by biological age, significant performance changes are only observed along the ‘Maturation’ axis within the age-ranges of 12.5–14.5 and 13.5–15.5 years. **c** Processing Speed is determined by biological age, significant performance changes are only observed along the ‘Maturation’ axis within the age-range of 11.5–13.5 years, although the impact of experience can also be observed at a smaller extent. **d** Perceptual Reasoning is not affected significantly by biological or chronological age within the investigated age-ranges. The final version of the figure was generated by Adobe Illustrator CC 23.02.2 (64-bit).

Partial correlation and linear regression analyses both demonstrate that CA and BA selectively impact on different cognitive domains. Although overall performance on WISC-IV is determined both by CA and BA, the abilities reflected by the indices of the WISC-IV are either affected by CA or BA, suggesting that individual domains are either experience or maturation determined. However, as we have seen in Fig. 2b,c, CA and BA effects might depend on the actual age-range, and developmental windows may shift also for different cognitive abilities. In order to scrutinize the strong, age-specific selective effects of CA and BA that are suggested by Tables 1 and 2 in terms of age-specificity, we continued with the graphical analysis presented in Fig. 2a. The results for the Verbal Comprehension, Working Memory, Processing Speed and Perceptual Reasoning indices are shown in Fig. 3.

Pearson correlation was calculated between each WISC index and BA and/or CA within each 1-year wide BA/CA group (Supplementary Table S6). The correlations were Holm–Bonferroni corrected for multiple comparisons. We used one-way ANOVA to look for significant differences between the lower and upper bins within the particular BA/CA age-band with significant correlations (Supplementary Table S7). Coloured arrows in Fig. 3 indicate those BA/CA age-bands where significant differences were found.

Figure 3a clearly shows that the Verbal Comprehension index of WISC-IV is determined by experience significantly at a constant 13.5 years of BA (73.3% ± 1.3 SE vs. 82.2% ± 1.2 SE; p < 0.001), while there is no significant maturity effect (76.4% ± 2.0 SE vs. 74.5% ± 3.2 SE; p = 0.591). Working Memory (Fig. 3b) on the other hand is BA dependent at a constant 13.5 and 14.5 years of CA (62.7% ± 1.4 SE vs. 68.3% ± 1.2 SE; p = 0.007 and 65.6% ± 2.8 SE vs. 75.6% ± 2.2 SE; p = 0.017, respectively), while there is no significant effect of experience (65.9% ± 2.0 SE vs. 65.6% ± 2.8 SE; p = 0.923 and 68.3% ± 1.2 SE vs. 79.2% ± 2.1 SE; p = 0.729, respectively). Processing Speed is showing a significant BA effect at a constant 12.5 CA (59.1% ± 1.8 SE vs. 65.0% ± 1.0 SE; p = 0.008), while the effect of experience does not reach significance (58.6% ± 2.2 SE vs. 63.9% ± 1.9 SE; p = 0.079, Fig. 3c). Perceptual Reasoning (Fig. 3d), corresponding to our analysis in Tables 1 and 2, does not seem to show any relevant BA or CA effects) within the investigated age-ranges.

## Discussion

We combined ultrasonic bone age estimation and a careful selection of participants according to their pubertal maturity levels to uncover the distinct effects of biological and chronological age on cognitive abilities. Unlike previous studies that employ less efficient control of pubertal maturity, our design allows for a clear-cut dissociation of the effects of maturity versus experience. By comparing the cognitive performance of participants with average, advanced and delayed skeletal maturity we revealed that overall cognitive performance is determined both by biological and chronological age. With respect to different broad cognitive abilities, we found that biological and chronological age selectively and independently influence them: among adolescents of the exact same chronological age, more mature individuals have higher memory capacity and processing speed, while among adolescents of the same maturity, those with higher chronological age have better verbal abilities. Our design also allowed for a detailed exploration of the shifting developmental windows of different cognitive abilities: we revealed age-specific effects with processing speed being affected by maturity levels at an earlier chronological age than working memory capacity.

With respect to the age-specific effects of maturity on different cognitive abilities, Working Memory and Processing Speed Indices of the WISC-IV stand out in our study. In addition to both being strongly correlated with the level of physical maturity, processing speed is affected by biological age earlier (12–13 years, Fig. 3c) than working memory (13–15 years, Fig. 3b). Although a strong association between processing speed and working memory during development has been asserted^42^, the picture is confounded unless controlling for age and maturity independently. The largest available population study including these variables is the Adolescent Brain Cognitive Development (ABCD) Study (https://abcdstudy.org). At its current stage, ABCD provides a conclusive analysis on mostly prepubertal 10-year-old children demonstrating that processing speed and working memory are not related at this age^43^. Our results corroborate this finding revealing the difference in the maturational timing of the two behavioural measures, and also support an interpretation emphasizing their diverse neural background. While processing speed is generally associated with white matter volume^44,45^, working memory is more specifically related to the late maturing or frontoparietal core network^46–49^.

Although working memory (Fig. 3b) seems to fulfil our predictions with respect to an exclusively maturation-determined (Fig. 1c) developmental process, verbal comprehension (Fig. 3a) exhibits a largely experience-determined (Fig. 1c) process. In other words, we have demonstrated the independent effect of BA (but not CA) on working memory, and the individual effect of CA (but not BA) on verbal comprehension. It is important to note that we have not demonstrated that working memory is dissociated from verbal comprehension; in fact, the correlation between these broad abilities was 0.339 in our sample.

Our results on the Verbal Comprehension Index (Fig. 3a) and the related subtests (see Supplementary Tables S3 and S4 online) agree with previous results which indicate that the verbal skills the WISC measures are determined by age and schooling. For instance, unlike nonverbal abilities, performance on the verbal subtests keeps increasing in adulthood and peaks in middle to old age—in fact, of all the WISC subtests these are the ones to peak latest in life^33^. Additionally, previous studies that employed a methodology capitalizing on a possible discrepancy between a child’s chronological age and their school grade in order to dissociate the effect of schooling from other kinds of experience that improve with age, found that in children schooling has a particularly strong effect on individual differences in this ability^35^. Therefore, our conclusion that chronological age has a strong effect on the Verbal Comprehension Index is in accord with previous findings. The extent to which this effect is due to schooling in particular warrants further research.

In addition to the effect of chronological age, the lack of an effect of maturation is also important. While one could plausibly expect such an effect provided the cognitive basis of language development, the Verbal Comprehension Index of the WISC, despite its name, does not strongly tap linguistic cognition. The same factor has been interpreted as Gc (Comprehension-knowledge)^50,51^. Our results demonstrate that *g* is not a unitary construct, one that could meaningfully represent a single general ability measured by different tests and reflected by overall IQ. Rather, the differential effect of maturation in different cognitive abilities points to the importance of specific abilities, rather than *g* or overall IQ, and suggests that these abilities should be the basis of research on individual differences, as proposed by process overlap theory^52,53^.

The Perceptual Reasoning Index of WISC-IV (Fig. 3d) does not seem to show any relevant biological or chronological age effects within the investigated age-ranges. This is somewhat against expectations since perceptual development extends well into the teenage years as we have demonstrated earlier^54,55^. While lower-level perceptual functions are adult-like in the early years^56^, integrative processes of perceptual organization mature relatively late^54,55^. On the other hand, the canonical back to front progression of brain maturation^57–59^ with the corresponding functional developmental trajectories^8,59,60^ may suggest that the developmental window (Fig. 1c) for the perceptual functions tapped by the probes in the Perceptual Reasoning Index is at an earlier age than our investigated range. Another possibility is that the probes within this Index are less reliable measures of function than those within the other scales. Detailed psychophysical studies employing the suggested biological/chronological age dissociation paradigm might decide between these options. Although we have already dissociated the role of maturity and experience in early visual development^61^, it would be extremely important to do the same beyond childhood since later perceptual alterations might be at the core of behavioural dysfunction in several neurodevelopmental disorders^62–66^.

The findings presented here have great relevance for clinical neuroscience, especially with respect to the so-called “connectopathies” such as schizophrenia or autism. Puberty related hormonal influence on the excess elimination of cortical synapses (“pruning”) have been indicated to play a role in early onset schizophrenia with evidence for overpruning^67,68^, while adolescent underpruning has been indicated among the various age-specific anatomical alterations in autism^69–72^. Variation in puberty onset times seems to be related to eating disorders and depression, with early maturing adolescents having a greater risk to develop those^73,74^. Clearly, adolescence is an incredibly sensitive period of hormone-dependent brain organization. Since the manipulation of gonadal steroid levels is not an option in human research, direct evidence is generally difficult to obtain in this field. Therefore, the establishment of a reliable pubertal maturity indicator, and an associated neurotypical baseline of brain structure and function is definitely necessary for a better understanding of the pubertal remodelling of the human brain that might lead to efficient clinical interventions.

In general, human neurocognitive development has a uniquely protracted course and the details on factors that determine maturational trajectories of brain connectivity are only beginning to emerge^75^. The orchestrating function of pubertal hormones behind overall adolescent progress seems clear and assumes an explanatory role. Pubertal hormone levels are correlated with bone age^25–27^, time bodily growth^76^, cortical pruning^77,78^, and emotional^1,4^ and cognitive^79,80^ development in adolescence. The cascade of events during the pubertal transformation of a child into an adult is coordinated on individual timescales, and any attempt to assess adolescent brain and cognitive function requires the determination of the individual timescales. Emphasizing the importance of individual differences in neurodevelopment^1,12^ and parsing the genetic versus environmental influences on the human brain^81^ is among those pertinent ideas that should move the field forward.

The current project is constrained to study adolescent girls only which might appear as a limitation. However, it is unavoidable to make a choice if the primary purpose is to clearly dissociate the effects of maturation and experience on cognitive development. Involving both sexes would have made it difficult to carry out the more detailed analysis into the narrower temporal windows for BA or CA-related effects presented in Figs. 2 and 3. Since male pubertal onset is delayed by 1–1.5 years with respect to that of females^38,39,40^, this analysis could have only been performed relying upon a much larger sample. We chose to include girls because menarche age (see Supplementary Fig. S1 online) serves as valuable additional information confirming the validity of bone age assessments. Based on the conclusive results with girls that indicate a strong role of biological age in cognitive development one might assume that a similar effect will also be observed in boys. Indeed, we would expect that maturity is a relevant factor in the general cognitive development of boys as well, however, since female and male developmental trajectories are determined by very different gonadal hormone levels^4,82^, we also anticipate alternative trajectories in terms of specific cognitive abilities. Such trajectories have recently been indicated, e.g., in visuospatial processing^83^ and visual decision making^84^.

Another restriction we felt important to introduce is to only involve students at top-level high schools. Since we primarily sought to study the effect of maturity on adolescent development, large differences in socio-economic or socio-cultural backgrounds as well as the preselection processes introduced by schools would have confounded our data. We chose to include schools at the high end since entrance exam scores of accepted students vary the least in those. Based on the findings of the current study, we would expect to find meaningful discrepancies in cohorts with socio-economic or socio-cultural handicaps where sexual precocity is more frequent^3,38^.

## Conclusions

In conclusion, we have introduced bone age assessment into adolescent research to replace less reliable methods of pubertal maturity estimation. This method allowed us to disentangle the independent effects of chronological and biological age on adolescent cognitive abilities. Thereby, we compared cognitive performance of participants with average, advanced and delayed skeletal maturity and uncovered the striking impact of biological age on overall cognitive performance in addition to the known and expected effect of chronological age. With respect to the tested broad cognitive abilities, we discovered that more mature individuals within the same chronological age group have higher working memory capacity and processing speed, while those with higher chronological age have better verbal abilities, independently of their maturity levels. The paradigm introduced here is expected to fill a methodological niche for future studies on adolescent brain development, and the results already obtained will advance developmental neuroscience, school and clinical psychology as well as the study of the nature of intelligence and the determinants of individual differences in cognitive abilities.

## Methods

### Participants

Since median age at menarche is between 12.5–13.5 years of age in females^38,3^, and male pubertal onset lags behind by about 1–1.5 years^38–40^, it would have been difficult to include both sexes in the current study. Since our aim is to investigate whether biological age has a significant effect on cognitive development as a function of maturity, the delayed maturation of boys would have confounded the design described in Fig. 1a. Therefore, we only tested female participants (mean menarche age of the whole sample is 12.1 ± 0.9 SD years).

We recruited participants mainly by contacting schools and advertising online. Our participants were selected from high schools that belong to the top 10% in Hungary, according to a ranking based on university enrolment. Since Hungarian high schools accept pupils based on an entrance exam, and the highly ranked schools select students with the highest scores, it appeared relevant to limit the range of participating schools to avoid the confounding effect of school ranking. The top 10% seemed a good choice because these schools accept students uniformly with scores varying only slightly around maximal performance, while performance varies much more in high-average or average schools. Parental education in our selected sample, with 90% of the mothers and 88% of the fathers having a university degree, provides evidence for the similar background of our selected participants (see Supplementary Table S8 online).

Parents filled out a questionnaire on demographic data, parental education (see Supplementary Table S8 online), height, age at birth, number of siblings, residential area, time of menarche (see Supplementary Fig. S1 online), handedness, fine motor function, musical training, and visus. Parents also reported any history of developmental disorders, learning disability, neurological disorders, sleep disorder, attention deficit disorder or recent injury to the wrist area on the non-dominant hand. There was only one parent who reported attention deficit disorder in their child who was then excluded from further testing.

All participants went through an ultrasonic bone age screening procedure (see “Procedure”). Following the screening, we selected an approximately equal number of participants into the BA/CA defined bins as shown in Fig. 1b. In order to form discrete, 1-year-wide bins without any chance of overlap between the average, advanced and delayed categories, we defined four CA groups (Table 3), and constrained bin sizes in terms of BA: an average maturity bin is defined as CA − 0.5 ≤ BA ≤ CA + 0.5 years; an advanced maturity bin is defined as CA + 1.5 ≥ BA > CA + 0.5; a delayed maturity bin is defined as CA − 1.5 ≤ BA < CA − 0.5.

**Table 3:** Descriptive statistics of participants’ age.

CA groups	Maturity level	CA Mean (SD)	BA Mean (SD)
11–12 years	Advanced	11.48 (0.22)	12.45 (0.21)
	Average	11.63 (0.17)	11.60 (0.26)
12–13 years	Advanced	12.53 (0.25)	13.47 (0.24)
	Average	12.48 (0.28)	12.52 (0.24)
	Delayed	12.37 (0.28)	11.54 (0.22)
13–14 years	Advanced	13.68 (0.33)	14.53 (0.20)
	Average	13.51 (0.30)	13.47 (0.29)
	Delayed	13.49 (0.23)	12.53 (0.29)
14–15 years	Advanced	14.48 (0.32)	15.68 (0.35)
	Average	14.36 (0.24)	14.41 (0.28)
	Delayed	14.51 (0.23)	13.55 (0.31)

Descriptive statistics of the selected 117 participant cohort in terms of CA and BA. Maturity levels are grey-level-coded according to Fig. 1b. Each CA group starts at the birthday and ends just before the birthday of a participant (e.g.: 11.00–11.99 year). BA determines bin-sizes in a similar manner. The exact number of participants in each bin is presented in Fig. 1b.

Power calculations for the linear regression analysis indicated that a sample size near N = 120 was warranted in order to avoid type II error. In a pilot BA assessment study, we found that the percentage of adolescents with advanced or delayed maturation is around 20% in each group. Accordingly, we determined that the total number of participants to be invited for BA screening should be 300, with each CA group including 75 screened participants. With 300 participants screened, we predicted that there will be at least 10 participants within each bin. Therefore, 300 female students between the ages of 11 and 15 years were invited for BA screening. In order to eliminate the prospect of endocrinological complications, we excluded extremely advanced (BA > CA + 1.5, 21 girls, 7.0%) and extremely delayed (BA < CA − 1.5, 16 girls, 5.3%) participants from further testing. An additional 34 (11.3%) participants did not fit into any of our predetermined bins (falling just outside of the hexagons in Fig. 1b). 105 (35.0%) participants fell into average, 67 (22.3%) participants into advanced, and 57 (19.0%) participants into delayed bins of skeletal maturation. Out of the 229 participants falling into the predetermined bins, 53 girls have not accepted our invitation for further testing, and 59 girls were not invited back since we reached our predetermined sample size.

The final cohort described in Fig. 1b and in Table 3 consisted of 117 girls. These 117 girls participated in WISC-IV testing. All of them speak Hungarian as their mother tongue. 17 participants are left-handed and there is a mixed-handed participant. One of the participants got distracted during the administration of the Cancellation subtest, therefore their result on this subtest was discarded from the analysis.

The Hungarian United Ethical Review Committee for Research in Psychology (EPKEB) approved the study (reference number 2017/84). Written informed consent was obtained from all subjects and their parents. Participants were given gift cards (approx. EUR 15 value each) for their attendance. All research described in this paper was performed in accordance with the Declaration of Helsinki.

### Procedure

#### Bone age assessment

After body height (DKSH anthropometer, Switzerland Ltd, Zurich, Switzerland) and body weight (Seca digital scale) measurements, we assessed skeletal maturity (bone age) with an ultrasonic device (Sunlight BonAge, Sunlight Medical Ltd, Tel Aviv, Israel). Ultrasonic testing prevents children from being exposed to radiation in contrast to earlier, X-ray based methods, and at the same time, ultrasonic estimations highly correlate with X-ray estimations^29^. These features make ultrasonic screening optimal for research purposes. Ultrasonic bone age estimation is based on the changing acoustic conductivity of forearm bones during growth.

Ultrasonic bone age estimation was carried out at the school of the participants or at the Research Centre for Sport Physiology at the University of Physical Education, Budapest.

We performed measurements on the left hand and wrist region. Participants placed their arm on the armrest surface between the transducers. We adjusted the transducers to the forearm growth zone (to the connection of the distal epiphysis and diaphysis). Then, at the initial position, the device adhered to the forearm at a pressure of approximately 500 g and emitted ultrasound at a frequency of 750 kHz to the measurement site for each measurement cycle. One measurement cycle lasted about 20 s and was repeated five times. Between the measurement cycles, the transducers rose up 2 mm in the palm-back direction. The device estimates bone age (in years and months) by measuring the speed of sound (SOS) and the distance between the transducers, using algorithms based on gender and ethnicity^85^. The same person performed all measurements.

#### WISC-IV testing

Within a maximum of 3 months after the bone age assessment of each participant we administered 11 subtests of the Hungarian adaptation of the Wechsler Intelligence Scale for Children IV—WISC-IV^35^: Verbal Comprehension Index—Similarities, Vocabulary, Comprehension; Perceptual Reasoning Index—Block Design, Visual Puzzles, Matrix Reasoning; Working Memory Index—Digit Span, Letter-Number Sequencing; Processing Speed Index—Coding, Symbol Search, Cancellation. For the justification of subtest selection in the current study please see Supplementary information/WISC subtest selection. The administration of each subtest followed the standard testing and scoring procedure according to the test manual^86^.

WISC-IV subtests were administered in one session and scored according to the manual. Four trained school or clinical psychologists carried out the testing at the Laboratory for Psychological Research at PPCU. Scoring of the verbal comprehension subtests have subjective elements, therefore, we assessed interscorer reliability. Three cases were randomly selected from the sample and scored independently by all four psychologists. Intraclass correlation coefficient of interscorer reliability was 0.992.

### Statistics

We have carried out a step by step statistical analysis. First, we tested whether there is an independent effect of CA and/or BA on the overall cognitive performance and on the four main factors of WISC for the entire cohort. Where significant results were found we continued by a more detailed analysis to reveal the narrower temporal windows where major developmental events occur as a function of CA or BA. These were the actual steps of the analysis:

1. Partial correlations and multiple linear regressions—5 individual models predicting each specific ability and overall performance—were computed to investigate the independent effects of the two predictors (CA, BA). See Tables 1 and 2 of the main text, and Supplementary Tables S3 and S4 for more detailed results.
2. Since the two predictors have a correlation of r = 0.76 which is high, we have also carried out relative weight analysis (RWA) to see if the two predictors are tapping into the same variance or not (see Supplementary Table S5 online.)
3. Where regression was significant (and RWA demonstrated that the effect is genuine, not an artifact resulting from multicollinearity), we performed correlations between CA/BA and WISC-IV broad abilities and overall performance within 1-year-wide age-groups corresponding to the two orthogonal dimensions of Fig. 1a. Detailed correlations are in Supplementary Table S6. Figures 2a, and 3 (the coloured bin representations) summarize the correlation results: purple arrows indicate the age-ranges where BA, and green arrows indicate the age-ranges where CA had a significant effect after Holm–Bonferroni corrections for multiple comparisons.
4. We also performed one-way ANOVA within the age-ranges where correlations were significant after Holm–Bonferroni corrections, to see if the difference between delayed and advanced maturation groups within the CA and/or BA ranges is significant. The results of ANOVA are in Supplementary Table S7, and they are summarized in Fig. 2b, and in the line graphs of Fig. 3.

This multistep analysis not only shows whether there is an independent effect of, e.g., BA on Working Memory (partial correlation and regression), but it also indicates that the contribution of BA is about 78% (RWA analysis). We also learn that BA has a significant effect between 12 and 13-years-of-age on overall performance (Fig. 2a), and that there is a significant difference between delayed and advanced groups in Processing Speed within that temporal window, possibly accounting for the overall effect.

Statistical analyses were performed using IBM SPSS Statistics for Windows, Version 24.0 (Armonk, NY: IBM Corp.) and TIBCO STATISTICA 13.5.0.17 software (TIBCO Software Inc., 2018). Relative Weight Analysis was performed using RWA-Web App^87^.

## Supplementary Information

Supplementary Information.

## Data Availability

Demographic, anthropological and psychometric datasets are available at the Open Science Framework (OSF) platform at this address: https://osf.io/ys679.
