# Supplementary material for: Ultrasonic bone age fractionates cognitive abilities in adolescence
Source: Sci Rep. 2022 Mar 29;12:5311. doi: 10.1038/s41598-022-09329-z (PMC8964807; doi:10.1038/s41598-022-09329-z)
Supplement: Supplementary file 1 — Supplementary Information. [file 41598_2022_9329_MOESM1_ESM.pdf]

# Supplementary information

# for

# Ultrasonic bone age fractionates cognitive abilities in adolescence

Ilona Kovács*[,a,b,c], Kristóf Kovács[d], Patrícia Gerván[a,b], Katinka Utczás[e], Gyöngyi Oláh[a,b], Zsófia Tróznai[e], Andrea Berencsi[f], Hanna Szakács[a], & Ferenc Gombos[a,b]

[a] Laboratory for Psychological Research, Pázmány Péter Catholic University, Budapest, 1088 Hungary
[b] Adolescent Development Research Group, Hungarian Academy of Sciences - Pázmány Péter Catholic University, Budapest, 1088 Hungary
[c] Institute of Cognitive Neuroscience and Psychology, Res. Centre for Natural Sciences, Budapest, 1117 Hungary
[d] Institute of Psychology, ELTE Eötvös Loránd University, Budapest, 1075 Hungary
[e] Research Centre for Sport Physiology, University of Physical Education, Budapest, 1123 Hungary
[f] Institute for the Methodology of Special Needs Education and Rehabilitation, Bárczi Gusztáv Faculty of Special Needs Education, Eötvös Loránd University, Budapest, 1097 Hungary

* corresponding author: Ilona Kovács, Laboratory for Psychological Research, Pázmány Péter Catholic University, 1 Mikszáth sq., Budapest, 1088 Hungary, e-mail: dr.ilona.kovacs@gmail.com

**Supplementary Figure 1: The association between menarche age and pubertal maturity assessed by ultrasonic bone age.**

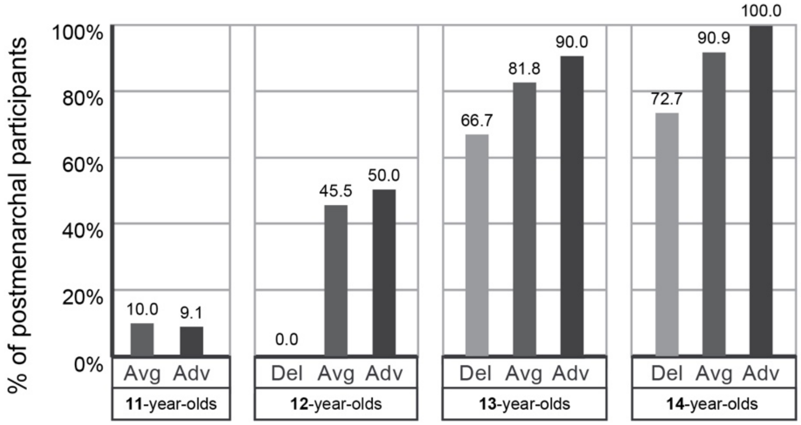


| Age (year) | Menarche age (year) Mean ± SD |
|---|---|
| All | 12.12 ± 0.93 |
| 11 | 11.45 ± 0.01 |
| 12 | 11.64 ± 0.45 |
| 13 | 12.14 ± 0.78 |
| 14 | 12.33 ± 1.15 |

The small table shows mean menarche age for four chronological age groups between 11 to 14 years of age. Mean menarche age increases with age as a larger proportion of girls becomes postmenstrual. The bar-graph show the percentage of participants who are already postmenstrual within the bone age determined maturational groups (see Figure 1.c of the main text), for each chronological age-group independently. There is an association between bone age determined maturity and the percentage of postmenstrual girls. More advanced maturity involves a higher percentage of postmenstrual participants. Notice that bone age defined maturity levels cannot be replaced by menarche age because this information is only available for postmenstrual girls, and there is no information on those who are not menstruating yet. However, the association presented in the figure provides support for bone age being a proper proxy for pubertal maturation.

**Supplementary Table 1: Descriptive statistics.**

| | M | SD | Range | N |
|---|---|---|---|---|
| Overall Performance | 71.34 | 4.89 | 60.15 - 80.84 | 117 |
| Verbal Comprehension | 75.01 | 8.13 | 53.24 - 90.02 | 117 |
| Perceptual Reasoning | 79.18 | 6.48 | 60.99 - 91.89 | 117 |
| Working Memory | 65.29 | 7.41 | 48.54 - 84.06 | 117 |
| Processing Speed | 63.81 | 7.22 | 43.94 - 78.37 | 117 |

| | M | SD | Range | N |
|---|---|---|---|---|
| VC – Similarities | 72.44 | 8.94 | 50.00 - 90.91 | 117 |
| VC – Vocabulary | 81.77 | 9.69 | 57.35 - 98.53 | 117 |
| VC – Comprehension | 70.82 | 9.39 | 50.00 - 90.48 | 117 |
| PR – Block Design | 80.74 | 11.39 | 44.12 - 98.53 | 117 |
| PR – Visual Puzzles | 74.88 | 8.86 | 57.14 - 100.0 | 117 |
| PR – Matrix Reasoning | 81.93 | 8.60 | 57.14 - 97.14 | 117 |
| WM – Digit Span | 60.12 | 10.06 | 37.50 - 87.50 | 117 |
| WM – Letter -Number Sequencing | 70.46 | 6.86 | 53.33 - 90.00 | 117 |
| PS – Coding | 54.30 | 9.56 | 29.41 - 78.15 | 117 |
| PS – Symbol Search | 55.88 | 8.66 | 33.33 - 75.00 | 117 |
| PS – Cancellation | 81.39 | 9.83 | 50.74 - 97.06 | 116 |

Descriptive statistics of WISC-IV broad abilities and overall performance (upper panel), and individual subtests. M = mean, SD = standard deviation (lower panel). VC = Verbal Comprehension, PR = Perceptual Reasoning, WM = Working Memory, PS = Processing Speed

## Supplementary Table 2: Correlations for the four factors of WISC-IV.

| | Chronological Age | 95CI | p | Biological Age | 95CI | p |
|---|---|---|---|---|---|---|
| Overall Performance | **.627** | **.514 - .726** | **<.001** | **.643** | **.533 - .739** | **<.001** |
| Verbal Comprehension | **.495** | **.343 - .643** | **<.001** | **.410** | **.246 - .573** | **<.001** |
| Perceptual Reasoning | **.306** | **.124 - .472** | **.001** | **.275** | **.105 - .432** | **.003** |
| Working Memory | **.271** | **.091 - .440** | **.003** | **.441** | **.310 - .558** | **<.001** |
| Processing Speed | **.534** | **.380 - .665** | **<.001** | **.582** | **.443 - .694** | **<.001** |

p < .01 p < .001

Correlations between chronological age / biological age and WISC-IV broad abilities and overall performance. 95CI = 95% confidence interval

## Supplementary Table 3: Partial correlations for the subtests of WISC-IV.

| | Chronological Age | 95CI | p | Biological Age | 95CI | p |
|---|---|---|---|---|---|---|
| VC – Similarities | **.209** | **.039 -.370** | **.025** | .077 | -.094 -.248 | .415 |
| VC – Vocabulary | **.246** | **.083 -.399** | **.008** | .067 | -.125 -.258 | .479 |
| VC – Comprehension | **.336** | **.182 -.488** | **<.001** | .016 | -.171 -.200 | .867 |
| PR – Block Design | .103 | -.090 -.323 | .272 | .140 | -.062 -.328 | .136 |
| PR – Visual Puzzles | .109 | -.073 -.272 | .246 | -.097 | -.277 -.091 | .301 |
| PR – Matrix Reasoning | .100 | -.059 -.273 | .289 | .079 | -.109 -.265 | .399 |
| WM – Digit Span | -.127 | -.313 -.075 | .177 | **.362** | **.210 -.500** | **<.001** |
| WM – Letter - Number Sequencing | -.035 | -.216 -.153 | .707 | **.270** | **.095 -.432** | **.004** |
| PS – Coding | .116 | -.053 - .289 | .216 | **.288** | **.142 -.419** | **.002** |
| PS – Symbol Search | .170 | -.033 -.367 | .070 | **.208** | **.021 -.386** | **.025** |
| PS – Cancellation | .100 | -.090 -.303 | .290 | **.201** | **.049 -.351** | **.032** |

p < .05 p < .01 p < .001

Partial correlations between chronological age / biological age and WISC-IV subtests. The correlations represent the *independent* effect of chronological age and biological age, with the effect of the other kind of age controlled for. 95CI = 95% confidence interval. VC = Verbal Comprehension, PR = Perceptual Reasoning, WM = Working Memory, PS = Processing Speed

## Supplementary Table 4: Linear regressions for the subtests of WISC-IV.

| | | B | 95CI | beta | p | Model parameters |
|---|---|---|---|---|---|---|
| VC – Similarities | Chronological Age | **.027** | **.005- .048** | **.319** | **.016** | F(2,114) = 10.561, p < .001<br>R = .397<br>$R^2$ = .157 |
| | Biological Age | .007 | -.012- .027 | .097 | .458 | |
| VC – Vocabulary | Chronological Age | **.033** | **.010- .056** | **.361** | **.006** | F(2,114) = 12.923, p < .001<br>R = .430<br>$R^2$ = .185 |
| | Biological Age | .007 | -.014- .028 | .086 | .502 | |
| VC – Comprehension | Chronological Age | **.042** | **.020- .063** | **.474** | **< .001** | F(2,114) =18.124, p < .001<br>R = .491<br>$R^2$ = .241 |
| | Biological Age | .002 | -.018- .021 | .022 | .857 | |
| PR – Block Design | Chronological Age | .015 | -.014- .043 | .139 | .304 | F(2,114) = 6.83, p < .01<br>R = .327<br>$R^2$ = .107 |
| | Biological Age | .020 | -.005- .046 | .210 | .121 | |
| PR – Visual Puzzles | Chronological Age | The regression equation was not significant. | | | | F(2,114) = 0.572, p = .566 |
| | Biological Age | | | | | |
| PR – Matrix Reasoning | Chronological Age | .013 | -.009- .035 | .166 | .229 | F(2,114) = 4.008, p < .05<br>R = .256<br>$R^2$ = .066 |
| | Biological Age | .008 | -.012- .027 | .107 | .439 | |
| WM – Digit Span | Chronological Age | -.014 | -.038- .010 | -.152 | .243 | F(2,114) = 12.015, p < .001<br>R = .417<br>$R^2$ = .174 |
| | Biological Age | **.044** | **.022- .066** | **.519** | **< .001** | |
| WM – Letter - Number Sequencing | Chronological Age | -.001 | -.018- .016 | -.012 | .929 | F(2,114) = 8.556, p < .001<br>R = .361<br>$R^2$ = .131 |
| | Biological Age | **.021** | **.006- .037** | **.370** | **.006** | |
| PS – Coding | Chronological Age | .015 | -.006- .037 | .168 | .169 | F(2,114) = 21.13, p < .001<br>R = .520<br>$R^2$ = .270 |
| | Biological Age | **.031** | **.011- .050** | **.382** | **.002** | |
| PS – Symbol Search | Chronological Age | .019 | -.001- .039 | .240 | .056 | F(2,114) = 17.58, p < .001<br>R = .486<br>$R^2$ = .236 |
| | Biological Age | **.020** | **.002- .038** | **.279** | **.027** | |
| PS – Cancellation | Chronological Age | .013 | -.011- .037 | .140 | .290 | F(2,113) = 10.839, p < .001<br>R = .401<br>$R^2$ = .161 |
| | Biological Age | **.024** | **.002- .045** | **.285** | **.032** | |

p < .05 p < .01 p < .001

Multiple linear regressions for chronological age / biological age on WISC-IV broad abilities and overall performance, B = unstandardised coefficients, beta = standardised coefficients, 95CI = 95% confidence interval.VC = Verbal Comprehension, PR = Perceptual Reasoning, WM = Working Memory, PS = Processing Speed.

**Supplementary Table 5: Relative Weight Analysis results.**

| Relative Weight Analyses | | $R^2$ | Raw & rescaled relative weights | | Confidence interval for the raw weights | | Tests of statistical significance | | Predictor comparison | |
|---|---|---|---|---|---|---|---|---|---|---|
| | | | Raw Rel Weight | Rescaled rel weight | Lower bound | Upper bound | Lower bound | Upper bound | Lower bound | Upper bound |
| Overall performance | BA | 0.465 | 0.239 | 51.47 | 0.144 | 0.329 | 0.147 | 0.334 | -0.133 | 0.105 |
| | CA | | 0.225 | 48.53 | 0.142 | 0.307 | 0.14 | 0.313 | | |
| Working memory | BA | 0.201 | 0.157 | 78.41 | 0.078 | 0.252 | 0.034 | 0.233 | -0.204 | -0.035 |
| | CA | | 0.043 | 21.59 | 0.017 | 0.086 | -0.065 | 0.079 | | |
| Processing speed | BA | 0.358 | 0.207 | 57.97 | 0.113 | 0.302 | 0.109 | 0.302 | -0.165 | 0.058 |
| | CA | | 0.15 | 42.03 | 0.067 | 0.248 | 0.06 | 0.25 | | |
| Verbal comprehension | BA | 0.254 | 0.086 | 33.81 | 0.028 | 0.167 | 0.022 | 0.179 | -0.011 | 0.179 |
| | CA | | 0.168 | 66.19 | 0.073 | 0.267 | 0.073 | 0.276 | | |
| Perceptual reasoning | BA | 0.097 | 0.04 | 41.63 | 0.006 | 0.108 | -0.012 | 0.119 | -0.059 | 0.099 |
| | CA | | 0.057 | 58.37 | 0.009 | 0.142 | -0.008 | 0.155 | | |

Significant results

Predictors have a significant effect if the confidence interval of the tests of statistical significance does not contain zero. The effects of the predictors are significantly different if the confidence interval of the predictor comparison does not contain zero. The rescaled relative weights indicate that 78.4 % of the effect of these predictors can be ascribed to BA; 21.6% of the effect of these predictors can be ascribed to CA in the case of Working Memory. With respect to Verbal Comprehension, the rescaled relative weights indicate that 66.2 % of the effect of these predictors can be ascribed to CA; 33.8% of the effect of these predictors can be ascribed to BA. In the case of overall performance BA and CA have almost identical effects (51.5% vs 48.5%, respectively). The difference between relative weights was significant in the case of Working Memory.

## Supplementary Table 6: Correlations within 1-year-wide age-groups.

| Effect in MLR | Variable | Age (years ±0.5 years) | Mean | Std.Dv. | r2 | t | p | N | Constant | Slope | Constant | Slope | Significant after HB correction |
|---|---|---|---|---|---|---|---|---|---|---|---|---|---|
| BA | Overall performance | CA=12.5 | 0.695 | 0.036 | 0.265 | 3.342 | 0.002 | 33 | 4.210 | 12.033 | 0.418 | 0.022 | ✓ |
| | | CA=13.5 | 0.723 | 0.042 | 0.112 | 1.982 | 0.056 | 33 | 8.495 | 6.850 | 0.502 | 0.016 | |
| | | CA=14.5 | 0.754 | 0.039 | 0.119 | 1.949 | 0.061 | 30 | 8.341 | 8.072 | 0.541 | 0.015 | |
| | Working memory | CA=12.5 | 0.640 | 0.069 | 0.041 | 1.155 | 0.257 | 33 | 10.991 | 2.465 | 0.429 | 0.017 | |
| | | CA=13.5 | 0.640 | 0.062 | 0.196 | 2.748 | 0.010 | 33 | 9.469 | 6.217 | 0.216 | 0.032 | ✓ |
| | | CA=14.5 | 0.696 | 0.085 | 0.203 | 2.671 | 0.012 | 30 | 11.122 | 4.752 | 0.080 | 0.043 | ✓ |
| | Processing speed | CA=12.5 | 0.623 | 0.053 | 0.299 | 3.639 | 0.001 | 33 | 7.185 | 8.637 | 0.188 | 0.035 | ✓ |
| | | CA=13.5 | 0.657 | 0.054 | 0.091 | 1.763 | 0.088 | 33 | 10.286 | 4.815 | 0.402 | 0.019 | |
| | | CA=14.5 | 0.680 | 0.074 | 0.154 | 2.258 | 0.032 | 30 | 11.204 | 4.746 | 0.211 | 0.032 | X |
| CA | Overall performance | BA=12.5 | 0.694 | 0.044 | 0.211 | 2.924 | 0.006 | 34 | 6.147 | 9.170 | 0.407 | 0.023 | ✓ |
| | | BA=13.5 | 0.725 | 0.033 | 0.200 | 2.829 | 0.008 | 34 | 4.891 | 11.847 | 0.498 | 0.017 | ✓ |
| | Verbal comprehension | BA=12.5 | 0.739 | 0.069 | 0.105 | 1.941 | 0.061 | 34 | 9.471 | 4.116 | 0.419 | 0.026 | |
| | | BA=13.5 | 0.768 | 0.062 | 0.352 | 4.172 | 0.000 | 34 | 7.091 | 8.316 | 0.197 | 0.042 | ✓ |

p ≤ .05   p ≤ .01   p ≤ .001

Correlations between chronological age / biological age and WISC-IV broad abilities and overall performance within 1-year-wide age-groups corresponding to the two orthogonal dimensions of Figure 1.a. Those rows where the MRL column has BA, correlations are at fixed CA values. Those rows where the MRL column has CA, correlations are at fixed BA values. Note that we ran correlations only for those variable combinations where multiple linear regression (see Table 2. of the main text) was significant. Mean age in central bins was 12.5, 13.5, 14.5. Checkmarks in the last column indicate results still significant after Holm-Bonferroni correction. X in the last column indicates the result that was not significant after the correction.

**Supplementary Table 7: ANOVA results for the one-year-wide age-groups.**

| | Mean age of Avg (years) | | SS | df | MS | SS | df | MS | F | p |
|---|---|---|---|---|---|---|---|---|---|---|
| Overall performance | 12.5 | BA | **0.009** | **1** | **0.009** | **0.020** | **20** | **0.001** | **8.929** | **0.007** |
| | | CA | **0.011** | **1** | **0.011** | **0.041** | **21** | **0.002** | **5.456** | **0.029** |
| | 13.5 | BA | 0.004 | 1 | 0.004 | 0.046 | 20 | 0.002 | 1.674 | 0.210 |
| | | CA | **0.007** | **1** | **0.007** | **0.019** | **21** | **0.001** | **7.669** | **0.011** |
| Working memory | 13.5 | BA | **0.017** | **1** | **0.017** | **0.038** | **20** | **0.002** | **9.225** | **0.007** |
| | | CA | 0.000 | 1 | 0.000 | 0.141 | 21 | 0.007 | 0.010 | 0.923 |
| | 14.5 | BA | **0.047** | **1** | **0.047** | **0.115** | **17** | **0.007** | **6.949** | **0.017** |
| | | CA | 0.000 | 1 | 0.000 | 0.062 | 19 | 0.003 | 0.124 | 0.729 |
| Processing speed | 12.5 | BA | **0.019** | **1** | **0.019** | **0.044** | **20** | **0.002** | **8.553** | **0.008** |
| | | CA | 0.016 | 1 | 0.016 | 0.101 | 21 | 0.005 | 3.419 | 0.079 |
| Verbal comprehension | 13.5 | BA | 0.002 | 1 | 0.002 | 0.145 | 20 | 0.007 | 0.298 | 0.591 |
| | | CA | **0.046** | **1** | **0.046** | **0.041** | **21** | **0.002** | **23.871** | **0.000** |

$p \leq .05$ $p \leq .01$ $p \leq .001$

Where correlations were significant after Holm-Bonferroni correction in Supplementary Table 6., we carried out a one-way ANOVA analysis on the two extreme bins (without the middle bin) of the two independent dimensions of Figure 1.a. This analysis provides a view within narrower age-ranges at significant differences between delayed and advanced or between younger and older participants (see the line-graphs in Figures 2. and 3. in the main text).

**Supplementary Table 8: Distribution of parental education in the studied sample.**

| | Mother (n) | Mother (%) | Father (n) | Father (%) |
|---|---|---|---|---|
| Vocational school school | 1 | 0.9 | 4 | 3.5 |
| High school degree degree | 10 | 8.5 | 10 | 8.5 |
| University degree degree | 106 | 90.6 | 103 | 88.0 |
| Total | 117 | 100 | 117 | 100 |

To see whether parental education, as an indicator of socio-economic status, might be a contributing factor in the differences of cognitive development of the current sample, we carried out statistical analyses. The non-parametric independent-samples median tests showed that medians were the same both across bins and across the accelerated-average-decelerated dimensions (see Fig. 1a and Fig.1b of the main text for the definition of bins and dimensions) regarding parental education level (alpha=0.05, CI=95). The non-parametric independent-samples Kruskal-Wallis test showed that distribution of parental education was the same across bins. Independent-samples Kruskal-Wallis test also showed that parental education has the same distribution in accelerated, average and decelerated participant groups (alpha=0.05 and CI=95). Therefore, parental education does not contribute to differences in cognitive development in the current sample.

## WISC subtest selection

We purported to measure each broad ability with three tests. In the case of Verbal Comprehension and Perceptual Reasoning the ten core tests of the battery already include three tests of each. For Perceptual Speed, there are two core tests in the standard battery, and they can be supplemented with a third test, Coding, which we also administered. In the case of Working Memory there are two core tests in the standard battery and a third test, Arithmetic, is available as supplementary.

However, the status of the Arithmetic subtest is controversial. Upon examining the content of the test, it appears as a complex measure that taps on various domains at the same time: quantitative knowledge, quantitative reasoning, working memory, and even verbal comprehension. In fact, the Cattell-Horn-Carroll (CHC) model recognizes two different aspects of individual differences in math-related cognition: the separate broad ability factor 'Quantitative knowledge' reflects acquired quantitative or numerical knowledge, but not reasoning with such knowledge, while 'Quantitative reasoning', a narrow factor under the broad ability factor 'Fluid reasoning' represents reasoning with numerical material[1].

For it to be a better measure of Working memory, the publishers of the WISC-IV have substantially modified the Arithmetic test from previous versions: the math-knowledge load was reduced, and the working memory demands were increased[2]. Despite this, several studies investigating the factor structure of the WISC-IV found that it is still not a pure measure of working memory; it has been found that besides memory, it measures fluid reasoning[3,4] and/or crystallized intelligence/comprehension & knowledge[5,6], too. Even a study that confirmed Arithmetic as a measure of Working memory found that its factor loading is much smaller than of the core working memory subtests[7].

A study fitted a model in which the Arithmetic test was removed from the 4-factor structure of the WISC and was directly measuring the higher order *g* factor[8]. Such a model is equivalent to one in which a separate factor is added, of which Arithmetic is the single indicator. Therefore, such a factor is statistically redundant, but substantively it is more plausible to claim that Arithmetic is a measure of a fifth broad ability than a direct measure of *g*. Indeed, it appears that the main difficulty from a latent variable modelling perspective is that Arithmetic might be the single indicator of a fifth broad ability, only allowing for suboptimal models.

The manual did in fact consider such a 5-factor solution but discarded it because it did not improve model fit over the 4-factor solution. Yet, importantly from our perspective, the 5-factor solution with Arithmetic as the only indicator of the fifth factor did improve model fit in a particular age group: in 11–16-year-old children[2]. Since this is the exact age range we were targeting, after considering all the above evidence we decided against administering the Arithmetic test as a supplementary test of Working Memory.

# References


1. McGrew, K. S. CHC theory and the human cognitive abilities project: Standing on the shoulders of the giants of psychometric intelligence research. *Intelligence* **37**, 1–10 (2009).
2. Wechsler, D. *WISC-IV Administration and Scoring Manual (Wechsler Intelligence Scale for Children- Fourth Edition)*. (PsychCorp (Harcourt Assessment), 2003)
3. Keith, T. Z., Fine, J. G., Taub, G. E., Reynolds, M. R. & Kranzler, J. H. Higher order, multisample, confirmatory factor analysis of the Wechsler Intelligence Scale for Children - Fourth edition: What does it measure? *Sch. Psychol. Rev.* **35**, 108–127 (2006).
4. Flanagan, D. P. & Kaufman, A. S. *Essentials of WISC–IV assessment*. (Wiley, 2009).
5. Chen, H., Keith, T. Z., Chen, Y. & Chang, B. What does the WISC-IV measure? Validation of the scoring and CHC-based interpretative approaches. *J. Res. Educ. Sci.* **54**, 85-108. (2009).
6. Grégoire, J. *L'examen clinique de l'intelligence de l'enfant. Fondements et pratique du WISC-IV*. (Mardaga, 2009).
7. Golay, P., Reverte, I., Rossier, J., Favez, N. & Lecerf, T. Further insights on the french WISC-IV factor structure through bayesian structural equation modeling. *Psychol. Assess.* **25**, 496–508 (2013).
8. Schneider, W. J. What If We Took Our Models Seriously? Estimating Latent Scores in Individuals. *J. Psychoeduc. Assess.* **31**, 186–201 (2013).
